# Mr. Neville Chamberlain's Opportunity

**Published:** 1923-04

**Authors:** 


### CORRESPONDENCE.

[Correspondence on all subjects is invited, but we cannot be responsible for the opinions expressed by our correspondents, who must give name and address as a guarantee of good faith, but not necessarily for publication. Correspondents are reminded that conciseness greatly facilitates early insertion.]

### Mr. Neville Chamberlain's Opportunity.

*To the Editor of* THE HOSPITAL AND HEALTH REVIEW.

SIR,—The aims of your paper in regard to the public health, as set out in your New Year's issue, are excellent. And as everyone seems to be waking up at last to the fact that the housing question overshadows every other question in domestic politics, perhaps something will be accomplished.

Questions of housing and health must not, however, be allowed to become the playthings of politics. Every member of every Public Health Committee in the country has a responsibility in these matters which really cannot be put too high. It can be exercised only by constant and unremitting attention, by stinging public interest into activity in their own localities, by concerted pressure on Mr. Neville Chamberlain, and by whole-hearted co-operation with him if he rises to the occasion.

It has been said by unkind critics that many years ago Birmingham had a great citizen and has lived on his reputation ever since. Birmingham is a city with which Mr. Neville Chamberlain is not unfamiliar. There are housing conditions in that city to-day which are appalling; you have glanced at them in one of the articles in your series of interviews with local authorities. Mr. Neville Chamberlain has a double opportunity, that of revitalising the office of Minister of Health and that of showing that once more Birmingham can lead the way in local Government. He will, I am sure, have the backing of every "live" member of every public health authority if he gives them a courageous lead and keeps it up.

